# *Kcnn4* is a modifier gene of intestinal cystic fibrosis preventing lethality in the *Cftr*-F508del mouse

**DOI:** 10.1038/s41598-018-27465-3

**Published:** 2018-06-18

**Authors:** Amber R. Philp, Texia T. Riquelme, Pamela Millar-Büchner, Rodrigo González, Francisco V. Sepúlveda, L. Pablo Cid, Carlos A. Flores

**Affiliations:** 1 Centro de Estudios Científicos (CECs), Arturo Prat 514, Valdivia, Chile; 2 Universidad Austral de Chile, Valdivia, Chile; 3 Department of Translational Pulmonology, Translational Lung Research Center Heidelberg (TLRC), Member of the German Center for Lung Research (DZL), University of Heidelberg, Heidelberg, Germany

## Abstract

Nearly 70% of cystic fibrosis (CF) patients bear the phenylalanine-508 deletion but disease severity differs greatly, and is not explained by the existence of different mutations in compound heterozygous. Studies demonstrated that genes other than *CFTR* relate to intestinal disease in humans and CF-mouse. *Kcnn4*, the gene encoding the calcium-activated potassium channel K_Ca_3.1, important for intestinal secretion, is present in a locus linked with occurrence of intestinal CF-disease in mice and humans. We reasoned that it might be a CF-modifier gene and bred a CF-mouse with *Kcnn4* silencing, finding that lethality was almost abolished. Silencing of *Kcnn4* did not improve intestinal secretory functions, but rather corrected increased circulating TNF-α level and reduced intestinal mast cell increase. Given the importance of mast cells in intestinal disease additional double mutant CF-animals were tested, one lacking mast cells (*C-kit*^W-sh/W-sh^) and *Stat6*^−/−^ to block IgE production. While mast cell depletion had no effect, silencing *Stat6* significantly reduced lethality. Our results show that *Kcnn4* is an intestinal CF modifier gene partially acting through a STAT6-dependent mechanism.

## Introduction

Cystic fibrosis (CF) is an autosomal disease produced by mutations of the cystic fibrosis conductance regulator chloride channel (CFTR) gene^1^. The main characteristics of the CF syndrome are the impairment of epithelial cell transport of chloride and bicarbonate producing salt loss in sweat that can modify tissue homeostasis, and the accumulation of thick-viscous mucus and presence of acidic pH in the epithelial surface that can lead to severe obstruction and/or malfunction in organs like the intestine, airways and pancreas^2^. Inflammation is an important feature of CF that is accompanied of increased levels of inflammatory cells and cytokines^3,4^, but the origin of the inflammatory state in CF is matter of intensive investigation. Intriguingly, signs of inflammation occur more often in the absence of infection in patients^5^, a phenomenon also observed in animal models of the disease^6^.

A characteristic component of CF pathology is the compromise of the gastrointestinal system, with impaired function of pancreas, gallbladder, liver, and small and large intestine^7^. Disease in the small intestine affects 20% to 46% of patients and is the consequence of accumulation of poorly hydrated thick mucus in the lumen leading to intestinal obstruction in infants (meconium ileus) or adults (distal intestine obstructive syndrome)^8^. There is an increased level of inflammatory cytokines in the intestinal lumen of CF patients^9^ but it is not known whether this is a causative factor in the phenotype. Manifestation of intestinal obstruction is not always related to the type of *CFTR* mutation found in patients as is the case with pancreatic insufficiency^10^. Nevertheless, there is a significantly increased risk of developing meconium ileus in siblings of affected patients rather than in non-related patients^11–15^. Genetic factors affecting CF severity exert their effects through the maintenance of anion secretion in the intestine and this property is more likely to be shared between monozygous than dizygous twins^16^. All this evidence has supported the view that the severity and manifestation of intestinal disease in CF is dependent on the influence of modifier genes, that is to say on the existence of other genes that can modify the course of the disease independently of the type of mutation affecting the *CFTR* gene^17–20^. Even though intestinal CF disease in human patients and mice models displays different features, evidence supporting the existence of modifier genes has been also obtained using the mouse models for CF that happens to be affected by lethal intestinal obstruction mainly after weaning. Breeding experiments using a *Cftr*^−/−^ mouse show that survival is dependent on the mouse strain. Animals with increased survival displayed calcium-dependent chloride secretion that was absent in wild type mice, and a modifier locus significantly related with the occurrence of intestinal obstruction was mapped near the centromere of chromosome 7^21^. A modifier locus for meconium ileus (CFM1) was found in human chromosome 19q13^15^ in a region syntenic with the zone mapped in chromosome 7 of the mouse. Interestingly, the gene encoding calcium-activated potassium channel K_Ca_3.1 important in calcium activated intestinal anion secretion, is located in both syntenic regions^22,23^. Later work further explored the presence of CF modifier genes in human chromosome 19q13 and revealed an association of *KCNN4* polymorphic markers with meconium ileus in humans^24^, making it a strong candidate for CFM1. A separate study, however, found no evidence for linkage of meconium ileus to the CFM1 locus in a sample of CF twins and siblings^11^.

K_Ca_3.1 channels encoded by the *Kcnn4* gene are expressed in the intestinal epithelium and play a central role in calcium-activated anion secretion in mice and humans^25,26^. As the gene has been suggested as a putative modifier of CF severity in humans^24^, we predicted that increased activity of K_Ca_3.1 might contribute to compensate the failure in anion and fluid secretion leading to meconium ileus. It would follow that silencing the *Kcnn4* gene in CF mice should exacerbate the disease and lead to increased lethality. To test this hypothesis we have now bred double mutant animals carrying the most prevalent CF human mutation, the *Cftr*^*ΔF508/ΔF508*^ mouse, and *Kcnn4*^−/−^, a knock-out animal for the K_Ca_3.1 channel. Unexpectedly, silencing *Kcnn4* greatly improved the survival of CF mice, without alteration of their intestinal secretory function. Instead we observe that increased levels of circulating inflammatory cytokines and mast cell increase in the intestine of the CF animals were significantly reduced upon *Kcnn4* inactivation. Since K_Ca_3.1 channel inhibition reduces the release of inflammatory cytokines and affects the function of inflammatory cells^27–29^, we tested the role of the Th2 response in the occurrence of intestinal disease in the CF mouse. There is an increasing body of evidence implicating Th2 increased responses in CF disease^30^, and after silencing of the STAT6 regulator of Th2-driven immune response we observed a significantly reduced lethality in the CF animal. Our results support the contention that *Kcnn4* is indeed a modifier gene for intestinal CF, exerting its effect by downregulation of the immune response rather than through a direct effect on epithelial secretory function.

## Materials and Methods

### Animals

The *Kcnn4* null mouse^31^, the *Cftr*^tm1Eur^ mouse (hereafter named as *Cftr*^*ΔF508/ΔF508*^)^32^ and the *Cftr* conditional null mouse (*Cftr*^fl/fl^)^33^ were obtained from the respective laboratories of origin. The Tg(Vil-cre)20Syr (*Villin*^cre/−^), *Stat6*^−/−^ and the *C-kit*^*W-sh/W-sh*^ mice were purchased from the Jackson Laboratories. All animals were maintained for at least 20 generations in the C57BL/6 background. Double mutant animals were generated from double heterozygous mice. Animals were maintained with free access to food and water ad libitum. During all the trials we used strictly same chow (2019S, Teklad Diets, Madison, WI, USA) to avoid possible factors influencing intestinal distress in the animals. Unless specified animals used for experiments were 2–3 months old and, male and female mice were used as they became available. Animals were maintained at the Centro de Estudios Científicos (CECs) Specific-Pathogen-Free mouse facility and all experimental procedures were approved by CECs Institutional Animal Care and Use Committee and are in in accordance with relevant guidelines and regulations.

### Ussing chamber measurements

Stripped colon samples were placed in 0.1 cm^2^ surface area tissue holders and inserted as separators in modified Ussing chambers (Physiologic Instruments Inc., San Diego, CA, USA). Tissues were bathed with bicarbonate-buffered solution (pH 7.4), of the following composition (in mM): 120 NaCl, 25 NaHCO_3_, 3.3 KH_2_PO_4_, 0.8 K_2_HPO_4_, 1.2 MgCl_2_, 1.2 CaCl_2_ and 10 D-Glucose, gassed with 5% CO_2_–95% O_2_ and kept throughout the experiment at 37 °C. The transepithelial potential difference referred to the serosal side was measured using a VCC MC2 amplifier (Physiologic Instruments Inc., San Diego, CA, USA) under current-clamp mode. Short, 200 ms pulses of 10 μA were given with a 1 s period. The short-circuit currents were calculated using the Ohm’s law as previously described^34^. To determine the amplitude of sodium absorption 10 µM amiloride was added on the apical side. The cAMP-dependent chloride secretion was induced by adding 100 µM 3-isobutyl-1-methylxanthine (IBMX) plus 1 µM forskolin (FSK) and the cAMP response was inhibited by the basolateral addition of the KCNQ1/KCNE3 potassium channel inhibitor, chromanol 293B. Finally 100 µM carbachol was added on the basolateral side to induce calcium-activated anionic secretion. All drug additions were maintained up to the end of the experiment. The magnitude (∆Isc) of the currents was calculated as the difference after and before stimulation or inhibition as described previously^25^. The cAMP-induced chloride anionic secretion was calculated using the difference obtained after chromanol 293B instead of that elicited after forskolin/IBMX addition to avoid basal chloride secretion interference by tissue-released prostaglandins as described^35^.

### Cytokine determination

Serum samples were obtained from whole blood after retroorbital-plexus puncture. Cytokines were determined using the Cytometric Beads Array Th1/Th2/Th17 kit (BD Biosciences), following manufacturer’s instructions in a FACSCanto II flow cytometer (BD Biosciences) and FlowJo 7.6 software (TreeStar Inc., Ashland, OR, USA). For IgE measurements the BD Biosciences ELISA IgE detection kit was used following the manufacturer’s instructions. Absorbance was measured in a Perkin Elmer En Vision 2014 Multilabel Reader.

### Histology and ganglia perimeter measurement

Small intestine and skin samples were fixed in PFA 4% and sectioned to 4 µm thickness. Samples were stained with H&E, Toluidine Blue (for mast cells) and periodic acid Schiff (PAS, for mucopolysaccharides). Mast cell count was determined by counting mast cells per optical field (400X). We consider cells as near the gut associated lymphoid tissue (GALT) when the mast cell was no more than 1 optical field away from GALT. Determination of mast cell numbers was performed by three independent observers. Ganglia perimeter was determined in freshly isolated small intestine samples. We isolate the first four ganglia starting from the cecum towards ileum, measure their perimeter and considered the average of the four ganglia as one sample. Photos of the ganglia were obtained and perimeter was determined using Image J software.

### Bone marrow derived mast cell (BMMC) isolation and culture, proliferation and chemotaxis measurements

BMMCs were obtained as previously described^28^. Briefly, bone marrow cells were flushed from femurs, and cultured and differentiated to mast cells for 4 weeks in DMEM (Life Technologies) supplemented with 10% fetal bovine serum, 1% penicillin/streptomycin, 1% fungizone and 10 ng ml^−1^ IL-3 (PeproTech). Non-adherent cells in culture were transferred into new flasks containing fresh culture medium every week. After 4 weeks cell purity was evaluated by May-Grünwald Giemsa and Toluidine blue staining. Proliferation of BMMCs was determined using the CellTiter 96 Aqueous One Solution Cell proliferation Assay kit. Cells were stimulated with Stem Cell Factor (SCF) 10 ng ml^−1^ for 24 hrs. For chemotaxis experiments, upper chambers of the Transwell (8 µm pores) were seeded with 50,000 pre-sensitized BMMCs incubated with 300 ng ml^−1^ of anti-DNP IgE (Clone SPE-7) at 37 °C in a humid atmosphere (5% CO_2_) overnight, in DMEM supplemented with 10% fetal bovine serum, 1% penicillin/streptomycin and 1% fungizone. After that 10 ng ml^−1^ DNP-HAS antigen was added at the bottom chamber as the chemoattractant molecule and cells were counted after 3 hrs. The TRAM-34 inhibitor was added to the upper chamber.

### Statistical analysis

Comparisons have been made either with t-test, z-test or Mann-Whitney test as indicated. Survival analysis has been performed with the log-rank test using the Sigmaplot software.

## Results

### Generation of a double mutant *Cftr*^*ΔF508/ΔF508*^/*Kcnn4*^−/−^ mouse

**Figure 1:**
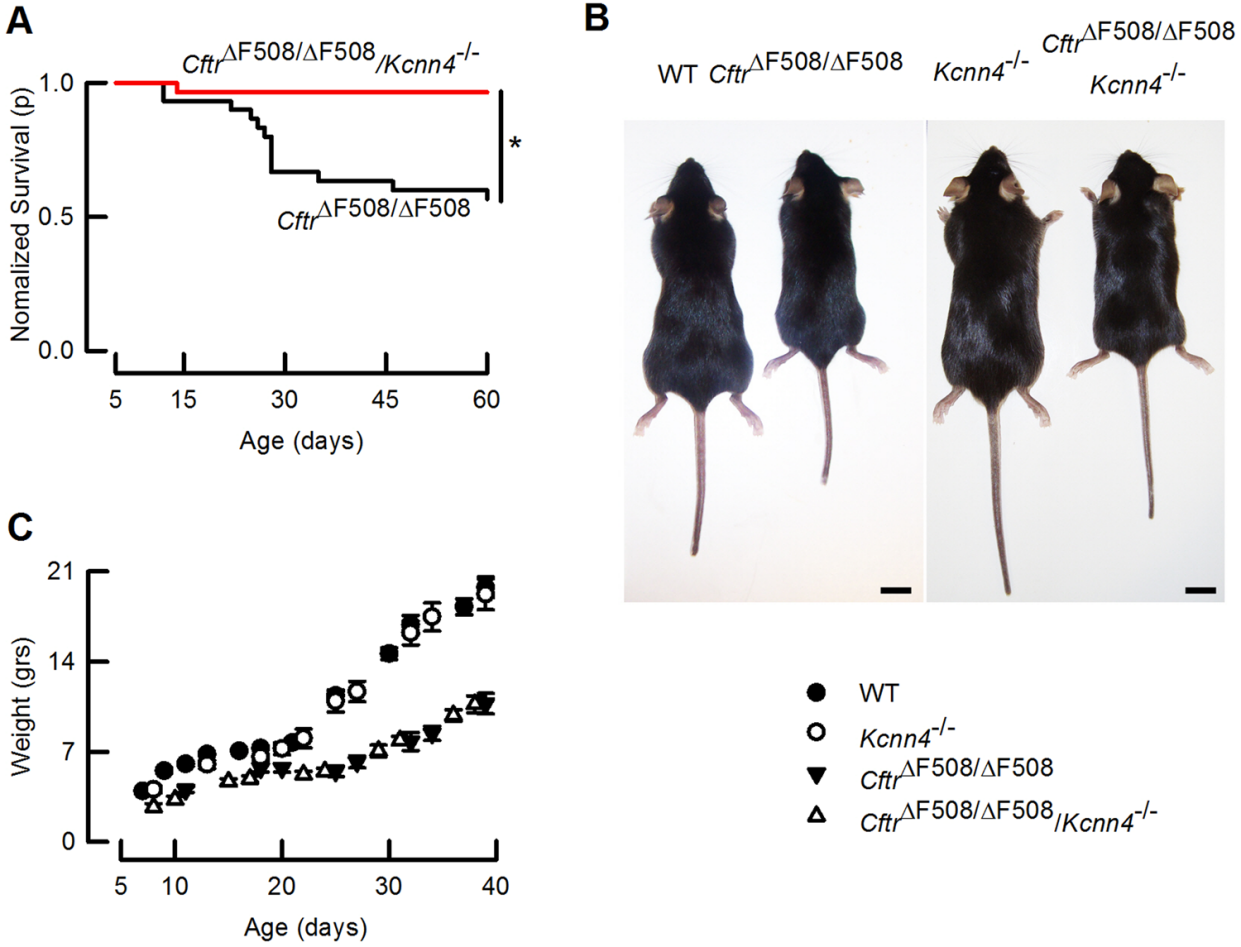
Increased survival of *Cftr*^*ΔF508/ΔF508*^ mice by additional *Kcnn4* inactivation. (**A**) Kaplan-Meier curves showing increased survival for the double mutant *Cftr*^*ΔF508/ΔF508*^/*Kcnn4*^−/−^ mouse compared to *Cftr*^*ΔF508/ΔF508*^. Log-Rank test, *indicates p = 0.001, n = 30 per group. (**B**) Photographs of animals of the four genotypes at 60 days old. Notice that the double mutant showed similar size to the CF mouse. Bar = 1 cm. (**C**) Time course for weight gain of animals of different genotypes. Break of the curves at day 21 corresponds to weaning. Values are given as mean ± S.E.M. with n = 8–9 per group. Calculated weight rates after and before weaning are summarized in Table 1.

The distribution of genotypes of animals obtained by mating double heterozygous animals for each genotype is compared with the expected Mendelian distribution in Supplementary Table 1. The data show that the genotypes generally fit with the expected distribution, except for the *Cftr*^*ΔF508/ΔF508*^ animals whose number is slightly lower. That could reflect the cannibalization of the dead pups by the mother before the genotyping and labelling could be performed (around day 5). Survival of mice homozygous for the *Cftr*^*ΔF508*^ mutation was increased when it was accompanied by the inactivation of *Kcnn4* (Fig. 1A). Lethality of the new phenotype at day 60 is 3.3% (1 out of 30) against 43.3% (13 out of 30) for the *Cftr*^*ΔF508/ΔF508*^ mouse. Most dead *Cftr*^*ΔF508/ΔF508*^ animals appeared after day 21, that corresponds to weaning and switch to solid food. Only one of the double *Cftr*^*ΔF508/ΔF508*^/*Kcnn4*^−/−^ mutants died before 60 days, but the body could not be recovered for a postmortem examination. We were able to recover 5 bodies of the *Cftr*^*ΔF508/ΔF508*^ animals, and the necropsy showed signs of small intestine rupture. No deaths were recorded for wild type or *Kcnn4*^−/−^ animals during the trial. We observed no gender-related lethality in the CF animals (5 dead out of 12 females and 8 dead out of 18 males, Differences in these proportions did not reach statistical significance as tested by z-test, P = 0.821). No gender-related survival was seen with the double mutants as 13 were females and 17 males, with only one death observed.

A decreased weight gain rate has been reported for CF patients and mouse models of the disease when compared with the normal population^36,37^. As shown in Fig. 1B, the *Cftr*^*ΔF508/ΔF508*^/*Kcnn4*^−/−^ and their *Cftr*^*ΔF508/Δ*F508^ littermates showed similar size. Growth rates expressed as weight gain over time are shown in Fig. 1C. The *Cftr*^*ΔF508/ΔF508*^ animals show a decreased weight gain after weaning compared with WT or *Kcnn4*^−/−^ mice. There is no evidence of weight gain recovery in the double mutants (See also Table 1).

**Table 1:** Summary of weight gain rates in mice.

	Up to day 20 (g day^−1^)	Days 21–40 (g day^−1^)
Wild type	0.26 ± 0.01	0.66 ± 0.04
*Kcnn4* ^−/−^	0.24 ± 0.02	0.69 ± 0.05
*Cftr* ^ΔF508/ΔF508^	0.23 ± 0.02	0.34 ± 0.04^a^
*Cftr*^ΔF508/ΔF508^/*Kcnn4*^−/−^	0.23 ± 0.03	0.35 ± 0.03^a^

Values are given as mean ± S.E.M., n = 8–9 animals per group. ^a^Indicates p < 0.05 compared to Wild type. Mann-Whitney test.

### Intestinal phenotype of the *Cftr*^*ΔF508/ΔF508*^/*Kcnn4*^−/−^ mice

**Figure 2:**
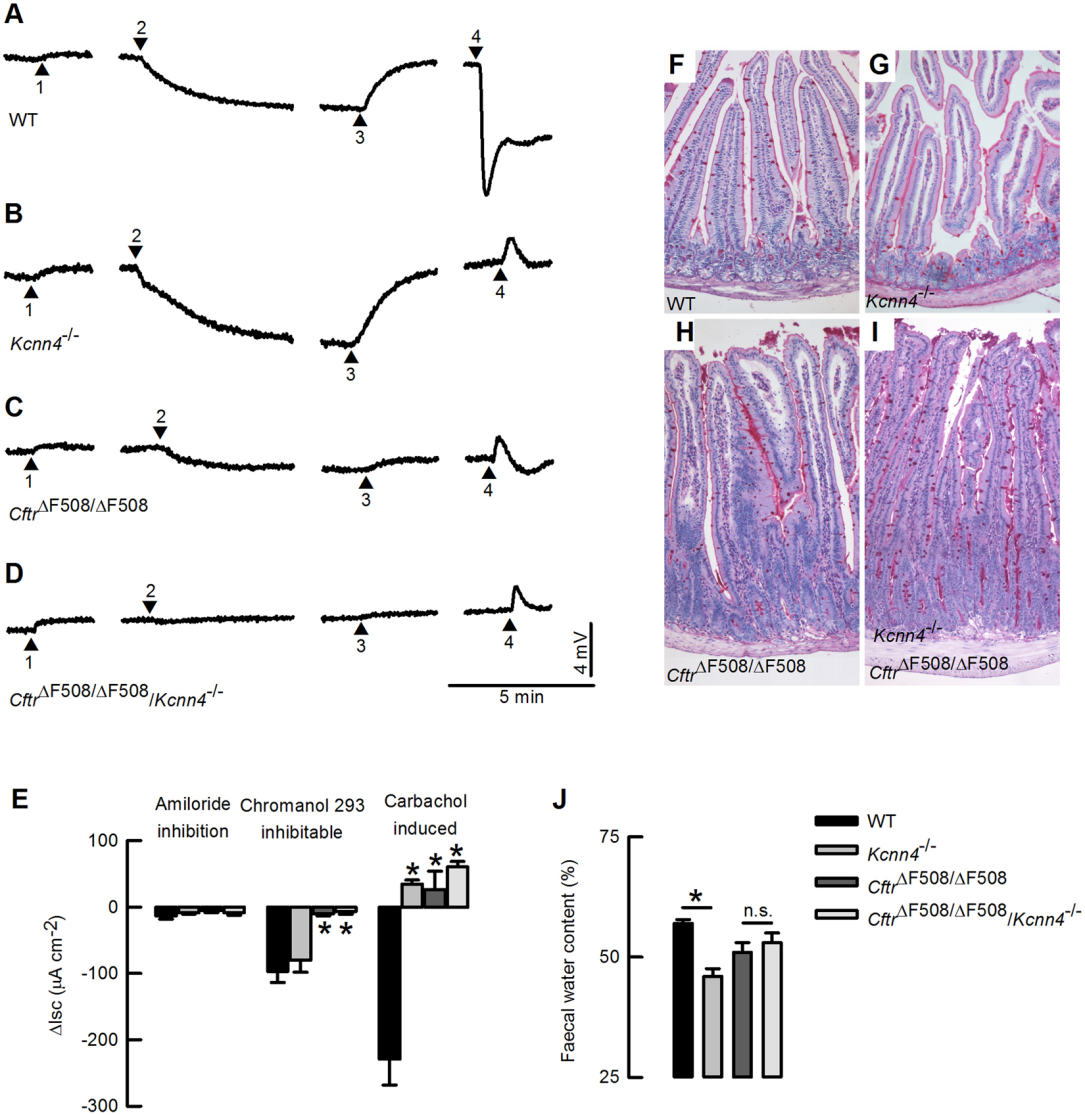
Effect of inactivation of *Cftr* and *Kcnn4* on electrophysiology and histology of intestinal epithelium. (**A**–**D**) Representative V_te_ traces of the four genotypes are presented. Number 1 indicates addition of 10 µM amiloride to the apical side. Number 2 indicates the addition of cAMP-increasing cocktail (100 µM IBMX+ 1 µM forskolin) to induce cAMP-activated anion secretion that is seen as a negative negative deflection in V_te_. cAMP-activated basolateral potassium channel KCNQ1/KCNE3 is inhibited with serosal 10 µM chromanol 293B (Number 3). Finally Ca^2+^-activated anion secretion is elicited by serosal addition of 100 µM carbachol (Number 4). All drugs are maintained constant after their corresponding addition to the bath solution. (**E**) Summary of calculated changes in short-circuit current under the different treatments. Values are given as means ± S.E.M., n = 3 for each group; *indicates p < 0.002 (unpaired Student’s t test) for comparisons with wild type (WT). (**F–I**) PAS staining of ileum histological sections showing mucus accumulation in the CF animals. Representative images of 5–6 samples for each group. **(J)** Summary of faecal water content in mice of the different genotypes. Values are given as means ± S.E.M., n = 5–7; *indicates p < 0.003 and n.s. is no statistical difference, unpaired Student’s t test.

The identification of genes coding for proteins responsible for alternative chloride conductances that are hypothesized to compensate for the transport defect brought about by CFTR inactivation, has been of high interest in CF research. We have explored the presence of novel chloride conductances in the colon of double mutant mice. Figure 2A–D show transepithelial voltage recordings of representative experiments for each genotype. We use a protocol to activate cAMP and calcium-activated anion currents independently by exposing the tissues to IBMX+Forskolin and Chromanol 293B or carbachol respectively. Consistent with previous observations in this model all *Cftr*^*ΔF508/ΔF508*^ animals have markedly reduced cAMP-activated and calcium-activated anion secretory currents^16,32,38^. More importantly, no novel calcium-activated anion conductance emerges in the double mutant animals, discarding a role for calcium-activated anion secretion as the mechanism behind the reduction in lethality. Positive currents, known to correspond to calcium-activated potassium secretion, are observed instead in tissues lacking K_Ca_3.1 or CFTR after carbachol stimulation and there is no calcium-activated chloride secretion induced by carbachol in *Kcnn4*^−/−^ tissue as previously shown^25^. Amiloride sensitive ENaC-mediated sodium absorptive currents were not affected. Figure 2E summarizes the calculated short-circuit currents for these experiments. It is apparent that amelioration of CF-associated lethality in the CF animals additionally deficient in K_Ca_3.1 is not associated to an altered ion transport phenotype. This would have been expected from the emergence of a chloride channel alternative to CFTR or the promotion of F508del expression into the plasma membrane.

**Figure 3:**
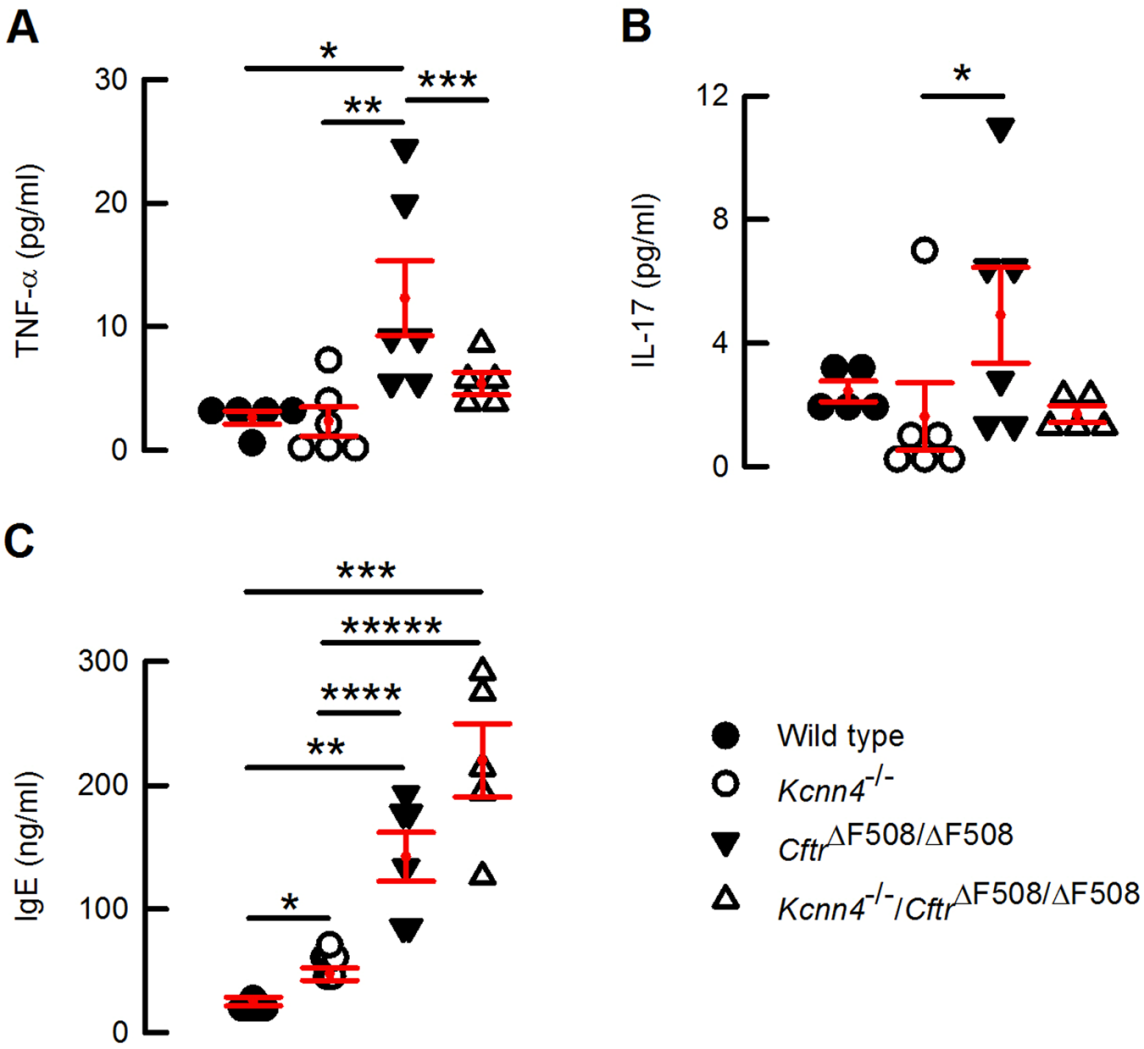
Effect of inactivation of *Cftr* and *Kcnn4* on cytokines and IgE in mouse serum. Summary of quantification for **(A)** TNF-α, **(B)** IL-17A and **(C)** IgE. Figures represent individual animals and red lines means ± S.E.M. Mann-Whitney test: (**A**) *indicates p = 0.004, **p = 0.009 and ***p = 0.03; (**B**) *indicates p = 0.041; (**C**) *indicates p = 0.002, **p = 0.002, ***p = 0.004, ****p = 0.002 and *****p = 0.004; n = 5–6 for each group.

The intestine from cystic fibrosis mouse models has been described as presenting mucus accumulation and changes in morphology that include enlargement of the villi^32,36,39,40^. We evaluate these features performing the periodic acid Schiff (PAS) staining. We found that the *Cftr*^*ΔF508/ΔF508*^ animals (Fig. 2H) indeed present mucus accumulation and the characteristic enlargement of villi when compared to wild type (Fig. 2F) and *Kcnn4*^−/−^ (Fig. 2G) intestines. Both abnormalities were also observed in the intestine from double mutants (Fig. 3I). Thus the silencing of *Kcnn4* in the *Cftr*^*ΔF508/ΔF508*^ mice did not change the previously reported intestinal histology of these CF animals.

Finally, we collected stool samples to determine its water content as a measure of the balance between absorption and secretion along the whole intestine. As shown in Fig. 2J the *Kcnn4*^−/−^ samples showed a significant decrease in faecal water content compared to wild type samples, confirming our previous observations. Nevertheless, the mutation in the *Cftr* gene does not greatly affects water content in the stools and additional silencing of *Kcnn4* does not disturbed hydration of the samples. In summary, our results showed that intestinal abnormalities normally found in the *Cftr*^*ΔF508/ΔF508*^ animals are not restored after *Kcnn4* silencing.

### Inflammatory cytokines are increased in CF mice

Increased inflammatory cytokines have been detected in CF mice and patients^41^. In view of the proposed role of K_Ca_3.1 in inflammatory cells^27–29^, we decided to measure the levels of circulating inflammatory and anti-inflammatory cytokines, and IgE in our animals to ascertain whether they were affected by additional *Kcnn4* inactivation. We found increased levels of IL-17A, TNF-α and IgE in the *Cftr*^*ΔF508/ΔF508*^ mouse (Fig. 3), with IL-6 and IL-10 remaining unchanged (not shown). Silencing of the K_Ca_3.1 channel has been demonstrated to decrease the release of cytokines, therefore we tested the effect of knocking out *Kcnn4* in the *Cftr*^*ΔF508/ΔF508*^ animals. TNF-α, but not IL-17A and IgE, were significantly reduced in the double mutants (Fig. 3), suggesting that the lack of K_Ca_3.1 diminishes the inflammatory cytokine TNF-α of CF animals. Other cytokines assayed, IFN-γ, IL-1β, IL-2 and IL-4, remained nearly undetected.

### Intestinal mast cell increase in the CF mice is reversed by *Kcnn4* inactivation

**Figure 4:**
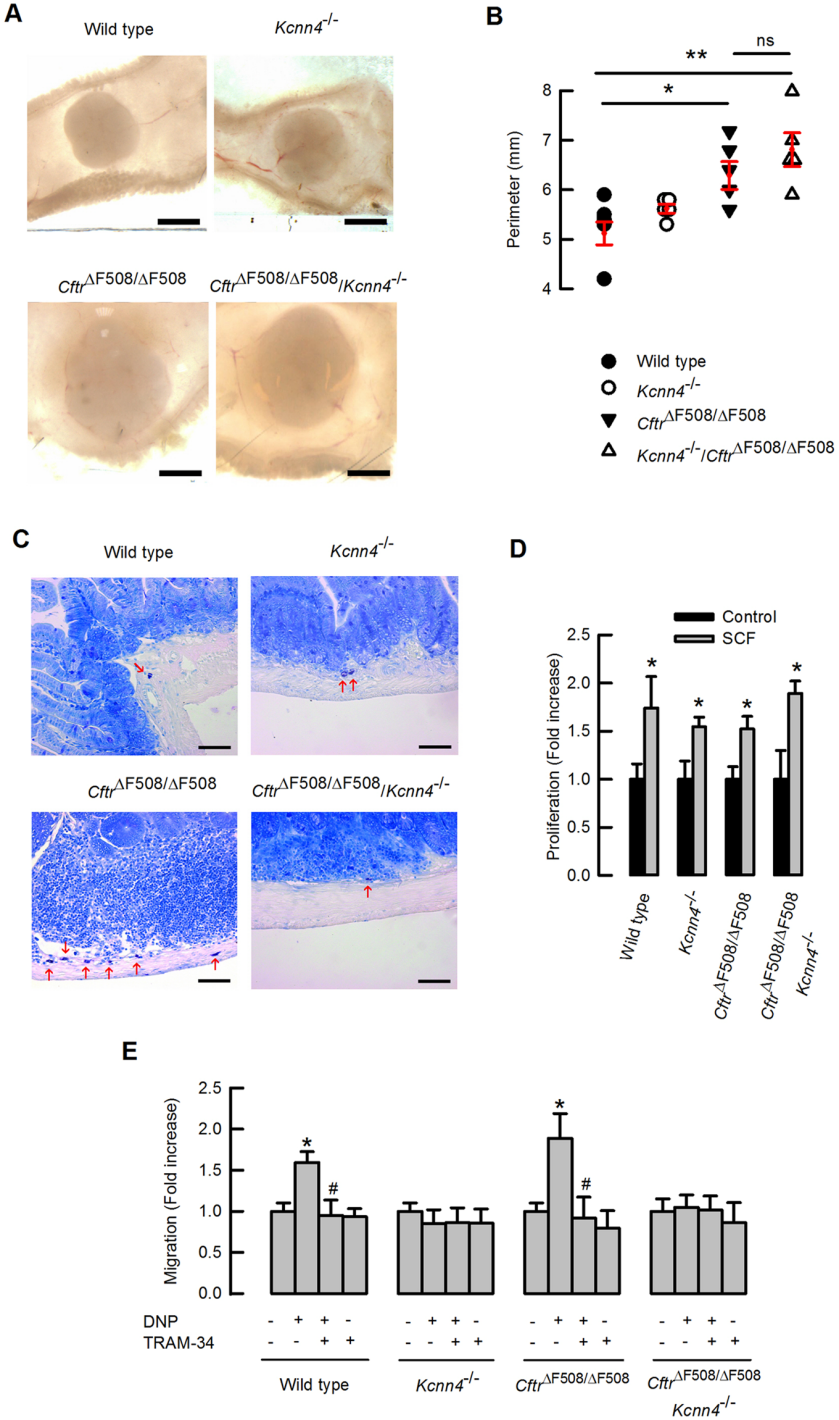
Intestinal mast cell increase in CF mice and reversal by simultaneous inactivation of *Kcnn4*. **(A)** Representative Peyer’s patches of each genotype, and **(B)** corresponding perimeter measurements. Figures represent individual animals and red lines means ± S.E.M. Mann-Whitney test: *indicates p = 0.016, **p = 0.008 and ns, non-significant difference; n = 5 for each group. **(C)** Toluidine blue staining of small intestine used to detect and quantify mast cells (red arrows). See Table 2 for values and statistics. **(D)** Proliferative response of mast cells in response to SCF is summarized. Values are given as mean ± S.E.M. n = 5. Paired Student’s t test: ^*^indicates p < 0.05 compared to the corresponding control. Mast cell chemotactic response to DNP and TRAM-34 effects are summarized in (**E**). Values are given as means ± S.E.M. Paired Student’s t test: *indicates p < 0.05 compared to the respective control. ^#^Indicates p < 0.05 compared to DNP effect, n = 6 for each genotype.

While performing animal dissections, we noticed that Peyer’s patches in the *Cftr*^*ΔF508/ΔF508*^ mouse appear bigger in size (Fig. 4A). Enlargement of Peyer’s patches is usually observed under intestinal inflammatory response^42^. Ganglia isolated from *Cftr*^*ΔF508/ΔF508*^ animals have a significantly larger perimeter than those of the wild type or *Kcnn4*^−/−^ animals. The enlargement, however, was maintained in the double mutants (Fig. 4B).

Histological analysis of toluidine blue stained small intestine uncovered a significantly increased number of mast cells in *Cftr*^*ΔF508/ΔF508*^ tissues. Importantly, when the K_Ca_3.1 channel was absent the number of mast cells in CF mice was reduced to normal values (Fig. 4C and Table 2). Mast cells were preferentially located in the muscularis externa and submucosal connective tissue, especially surrounding the Peyer’s patches (GALT), but we did not notice mast cells invading the mucosa. Parallel count of mast cells in the skin of the animals (another common target tissue of mast cells) showed no differences, indicating that mast cell increase was exclusive to intestine in the CF animals (Table 2).

**Table 2:** Distribution of small intestinal mast cells.

	Mast cells/field (All fields)	Mast cells/field (GALT fields)	Mast cells/field (Other fields)	Mast cells/field (Skin)
Wild type	0.07 ± 0.03	0.35 ± 0.28	0.04 ± 0.02	11.5 ± 0.8
*Kcnn4* ^−/−^	0.10 ± 0.04	0.16 ± 0.12	0.10 ± 0.05	12.3 ± 2.0
*Cftr* ^ΔF508/ΔF508^	0.41 ± 0.18^a^	1.46 ± 0.04^b,c^	0.14 ± 0.05	12.2 ± 1.2
*Cftr*^ΔF508/ΔF508^/*Kcnn4*^−/−^	0.18 ± 0.03	0.40 ± 0.19	0.15 ± 0.05	12.1 ± 0.4
*Cftr*^fl/fl^/*Villin*^Cre/−^	0.06 ± 0.02	0.20 ± 0.05	0.04 ± 0.02	± 0.5
Wild type	0.08 ± 0.04	0.40 ± 0.30	0.03 ± 0.02	13.2 ± 3.0
*Stat6* ^−/−^	0.08 ± 0.03	0.18 ± 0.09	0.04 ± 0.01	12.0 ± 2.0
*Cftr* ^ΔF508/ΔF508^	0.26 ± 0.07^d^	1.20 ± 0.32^e^	0.10 ± 0.06	13.8 ± 0.8
*Cftr*^ΔF508/ΔF508^/*Stat6*^−/−^	0.19 ± 0.08	0.69 ± 0.29	0.06 ± 0.02	13.6 ± 1.8

GALT: Gut associated lymphoid tissue.

Values are given as mean ± S.E.M. Unpaired Student’s test.

^a^Indicates p = 0.008,

^b^p = 0.032,

^d^p = 0.032 and

^e^p = 0.032 vs corresponding wild type group.

^c^Indicates p = 0.032 vs *Cftr*^ΔF508/ΔF508^/*Kcnn4*^−/−^; 80 to 120 400x optical fields per animal were analysed and n = 4–5 animals per group were used.

Mast cells can act as key regulators of inflammation in the intestine and their augmented numbers in the tissue could be due to local increased proliferation or to migration in response to local chemotactic cues^43,44^. Mast cells express CFTR and K_Ca_3.1 channels^29,45^ and inactivation of these channels might be expected to affect their function. We have tested whether the observed mast cell increase was related to functional changes in proliferation or migration in CF mice using mast cells differentiated from bone marrow. As shown in Fig. 4D, mast cell proliferation was increased in response to stem cell factor (SCF) in a similar manner in all four genotypes. Chemotactic response of mast cells to antigenic DNP was also evaluated. Cells sensitized with anti-DNP IgE migrate in response to DNP only when K_Ca_3.1 is present. In addition, chemotaxis was hampered when K_Ca_3.1 was blocked by the specific inhibitor TRAM-34 (Fig. 4F). CFTR mutation by itself did not alter the chemotactic response of mast cells to DNP (Fig. 4F). It appears therefore that changes in the ability of mast cells to respond to chemoattraction might be responsible for their reduction in CF mice lacking K_Ca_3.1.

It has been demonstrated that knockout of CFTR from intestinal epithelial cells induces the release of inflammatory cytokines^46^. To test whether mast cell chemoattracting molecule release specifically from intestinal epithelial cells is affected by *Cftr* silencing, we bred an intestinal-specific KO animal using a floxed *Cftr* mouse expressing the Cre-recombinase under the control of the intestinal-specific promoter villin. These animals are still affected by mucus accumulation and lethal intestinal obstructions but to a lesser degree that the *Cftr*^*ΔF508/ΔF508*^ *or Cftr*^−/−^ animals due to absence of intestinal anion secretion^47^ (Supplementary Figs 1 and 2). Histological analysis of tissues obtained from this intestinal-specific *Cftr* KO animal, the *Cftr*^*fl/fl*^*/Villin*^*cre/−*^ mouse, showed they harboured a normal number of mast cells in the small intestine (Table 2), discarding the participation of the epithelium in the recruitment of mast cells. We also observed that these animals present circulating levels of IgE (27.2 ± 2.3 ng/ml) similar to wild type (21.2 ± 1.7 ng/ml).

### Participation of mast cells and IgE in CF mouse lethality

**Figure 5:**
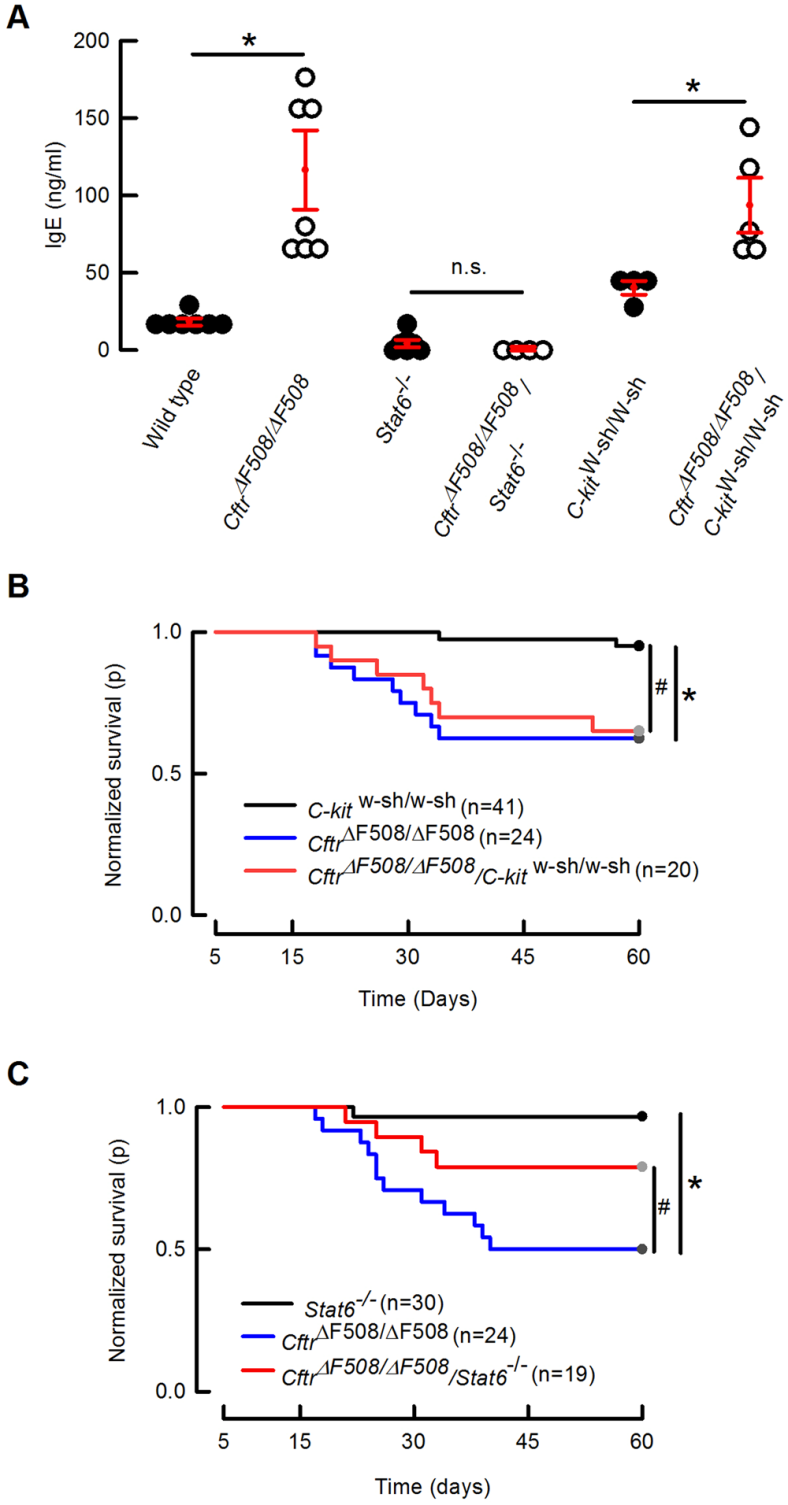
Effect of mast cell ablation or *Stat6* silencing on the survival of CF mice. **(A)** Circulating IgE levels were determined in the double and single mutant animals. Figures represent individual animals and red lines means ± S.E.M. Mann-Whitney test: *indicates p < 0.05 and n.s. (non-statistical difference) compared to the corresponding control. **(B)** Mast cell ablation in double mutant *C-kit*^*W-sh/W-sh*^*/Cftr*^ΔF508/^ΔF508 mice did not change the survival of these CF mice. Log-Rank test: *indicates p = 0.001 and ^#^p = 0.002. **(C)** Silencing of *Stat6* significantly reduced lethality of the CF animals. Log-Rank test: *indicates p = 0.0002 and ^#^p = 0.04.

To evaluate the participation of the IgE-mast cell axis in CF mouse lethality we bred two different double mutant CF animals, one devoid of mast cells using the *C-kit*^*W-sh/W-sh*^ mouse, and a second one using the *Stat6*^−/−^ animal that is unable to synthetize IgE. As observed in Fig. 5A the double mutant animal with no mast cells still exhibits increased IgE levels compared with the *C-kit*^*W-sh/W-sh*^ mouse. Figure 5B shows that the lethality of the *Cftr*^*ΔF508/ΔF508*^/*C-kit*^*W-sh/W-sh*^ mice is not different from that of the *Cftr*^*ΔF508/ΔF508*^ single mutant animals, and no gender related lethality was observed (Sup Table 2).

We next tested the role of IgE using the *Stat6*^−/−^ mouse. As observed in Fig. 5A the *Cftr*^*ΔF508/ΔF508*^/*Stat6*^−/−^ double mutant mouse shows an almost complete absence of IgE in the plasma. We also did not detect a significant reduction in the number of intestinal mast cells compared to what is seen in the *Cftr*^*ΔF508/ΔF508*^ mouse (Table 2), neither gender-related lethality (Sup Table 2). More importantly, silencing of *Stat6* significantly reduced the lethality of the *Cftr*^*ΔF508/ΔF508*^ mutant (Fig. 5C). Therefore it appears that mast cells themselves are not a determining factor in intestinal CF pathology. Silencing of *Stat6*, on the other hand, significantly contributes to reverting lethality in CF animals.

## Discussion

Cystic fibrosis phenotype heterogeneity cannot be directly attributed to the type of mutation affecting the gene encoding the CFTR protein. The current view also considers the influence of other genes and environmental factors that ultimately determine the severity of symptoms and organs affected in the patients. In the case of intestinal obstructions, it has been observed that the recurrence of meconium ileus is higher in affected siblings compared to that in unrelated patients, suggesting that genetic factors influence the manifestation of intestinal disturbances in CF. Studies designed to understand the genetic basis of the occurrence of meconium ileus have identified several genomic regions and single genes as putative modifiers in human CF patients^11,15,48–53^ and also in CF mouse models affected by intestinal obstruction^21,50,54,55^.

A region in the human genome and its syntenic region in the mouse genome have been postulated to harbour CF modifier gene(s) affecting the severity of meconium ileus in human CF patients and intestinal obstructions in CF murine models^15,21^. Located in this syntenic region, we found the gene coding for the calcium-activated potassium channel K_Ca_3.1^22,23^. A study of modifier genes for intestinal CF in human chromosome 19q13 has linked meconium ileus with polymorphic markers within the *KCNN4* gene^24^ making its product, the K_Ca_3.1, a strong candidate to modulate the severity of the effect of CFTR inactivation in intestinal function. Knockout of the K_Ca_3.1 protein in mice leads to significant reduction in water content of the faeces and to abolition of calcium-activated intestinal anion secretion^25^. The mechanism by which expression of the *Kcnn4* gene affects intestinal CF severity could therefore be related to the function of K_Ca_3.1 and its impact on calcium-activated chloride secretion. We have now tested this hypothesis through the generation of double mutant mice defective in CFTR and KCa3.1 expression.

*Cftr*^*ΔF508/ΔF508*^/*Kcnn4*^−/−^ double mutant mice are expected to show an increase in the occurrence of intestinal obstruction, and hence lethality with respect to single CF mice, due to an additional inhibition of calcium-activated secretion. Surprisingly, *Cftr*^*ΔF508/ΔF508*^/*Kcnn4*^−/−^ mice survived significantly better than the *Cftr*^*ΔF508/ΔF508*^ mice (Fig. 1A), consistent with the idea that *Kcnn4* is indeed a modifier gene for CF but suggesting an ameliorating effect of its inactivation rather than the aggravating effect expected from a straightforward mechanism involving its role in anion secretion. To understand how K_Ca_3.1 inhibition was able to reduce lethality of the CF animals we undertook studies of the potassium channel function in the intestine and the immune system, tissues known to be altered by *Cftr* mutation.

Defects observed in the intestine of CF animal models include deficient anion transport, mucus accumulation and goblet cell hyperplasia in most cases. These defects can also favour bacterial overgrowth and partially decreasing weight gain in the animals^21,32,36,39,40,47,56^. Nevertheless, analyses of growth rates and intestinal histology gave similar results for *Cftr*^*ΔF508/ΔF508*^ and *Cftr*^*ΔF508/ΔF508*^/*Kcnn4*^−/−^ mice, indicating no reduction of these alterations after *Kcnn4* silencing (Fig. 1C; Table 1). It has been observed that increased survival of a CF animal is related to calcium-activated anion secretion in the intestine. This is said to compensate for the absence of CFTR activity and in some cases is also accompanied with decreased mucus accumulation^21^. But again, calcium-activated anion secretion was not detected, faecal water content was not increased and mucus accumulation was still found on the *Cftr*^*ΔF508/ΔF508*^/*Kcnn4*^−/−^ animals (Fig. 2).

It has been demonstrated that CFTR, or its F508del mutant, and the K_Ca_3.1 channel proteins physically interact during their biogenesis in heterologous expression in HEK-293 cells^57^. A possible explanation for the decrease in lethality in our double mutant mice, therefore, is that elimination of K_Ca_3.1 might release intracellularly-retained mutant CFTR channels into the plasma membrane. We found no differences, however, in anion currents in the colon or faecal water content that could explain enhanced survival of the *Cftr*^*ΔF508/ΔF508*^/*Kcnn4*^−/−^, ruling out this hypothesis (Fig. 2). Additional inactivation of K_Ca_3.1 in CF animals did not affect key aspects of the intestinal CF phenotype, but clearly prevented intestinal obstruction-associated lethality. This indicates that K_Ca_3.1 inhibition rescues the CF intestinal phenotype most probably by affecting tissues other than intestinal epithelium.

Given that CF animals present an altered inflammatory response^6^ we looked for signs of this in the intestine and measured serum cytokines. We found increased levels IL-17A, TNF-α and IgE in the *Cftr*^*ΔF508/ΔF508*^ mouse (Fig. 3). Examination of the intestine showed enlarged Peyer’s patches and increased number of mast cells (Fig. 4A–C and Table 2). Silencing *Kcnn4* in the *Cftr*^*ΔF508/ΔF508*^ mouse reduced TNF-α and intestinal mast cells, but IgE and IL-17A levels and Peyer’s patch enlargement remained unchanged. These results indicate that K_Ca_3.1 inhibition can effectively reduce some inflammatory signals in the CF mice. Humans affected by CF present signs of intestinal inflammation^58^, and some of the observed immunological disturbances such as increased number of intestinal mast cells and increased IgE are also seen in mouse models with total CFTR deletion or lymphocyte-specific inactivation CFTR,^6,59^. Patients exhibit significantly higher levels of IgE after colonization by *Aspergillius fumigatus* when compared with non-CF individuals^60^. Altered distribution of mast cell subtypes, increased IL-17A and T_H_17 cells, and increased levels of TNF-α have all been reported in the lungs of CF patients^4,61–64^. Although, T_H_17 cells and IL-17A are extremely important for the maintenance of small intestine epithelial barrier function^65^, there is no evidence linking these or other inflammatory markers with the occurrence of meconium ileus. The tissue of origin for increased cytokine production and release in the *Cftr*^*ΔF508/ΔF508*^ animals remain to be determined.

Working with cultured mast cells we and others have observed that the genetic silencing or inhibition of K_Ca_3.1 channels impairs migration and release of granules, without affecting proliferation^29,66^. In this respect, impaired migration after K_Ca_3.1 inhibition can explain the reduction in mast cell increase in the CF intestine (Table 2). After being activated by IgE, mast cells can release a series of inflammatory mediators that have a profound impact on neighbouring tissues including intestine where, for example, epithelial permeability can be increased by proteases released from mast cells^67^. As it can be observed in the survival curves of Figs 1A and 5B,C, most of CF animals affected by lethal intestinal obstructions appeared around weaning at day 21. Increased intestinal permeability at weaning can be prevented with inhibitors of mast cell activation in pigs^68,69^. Since we found increased levels of IgE and mast cell increase in the CF animal, we thought that eliminating mast cells or IgE might improve CF mice survival. This was not the case as double mutant CF animals lacking mast cells displayed a similar lethality as the single CF mutant mice (Fig. 5B). Moreover, *Cftr*^fl/fl^/*Villin*^cre/−^ animals that do not show intestinal mast cell increase or increased IgE are still affected by increased lethality due to intestinal obstruction of lesser extent than for the *Cftr*^*ΔF508/ΔF508*^ or *Cftr*^−/−^ animals (Supplemental Fig. 1 and Hodges *et al*.^47^), suggesting a role for epithelial cells and additionally for immune cells of the intestine in the development of CF intestinal disease. Using the *Stat6*^−/−^ animals to eliminate IgE on the other hand, we observed a significant reduction in the CF-associated lethality (Fig. 5C). It is also important to note that the reduction of lethality obtained after *Stat6* silencing is smaller than that observed after *Kcnn4* silencing, indicating that K_Ca_3.1 channels are probably acting upstream of STAT6. However, it must be considered that STAT6 controls more than IgE production as it is a key factor in IL-4-triggered responses^70^. The results obtained here indicate that STAT6-dependent reduction of lethality compromises more than the IgE-mast cell axis, and must be related with a more profound disruption of IL-4-driven Th2 response in the CF mouse but also affecting CF lung disease in humans. For example, when *Cftr* is exclusively deleted from lymphocytes there is a spontaneous increase on serum IgE and increased release of IL-4, IL-5, IL-13 and IL-17 from CD4+ cells in the mouse^59^, while in CF patients Th2-driven immune response is frequently upregulated^30,71^. Whether alteration of Th2 response is at the core of CF disease is a question requiring further exploration. Interestingly, evidences for the expression of markers for autoinflammatory diseases in cystic fibrosis have been recently compiled^72^. For example, NLRP3-inflammasome activation and IL-1β release are found to be increased in the *Cftr*^−/−^ mouse lungs and human bronchial epithelial cells^73^. An imbalance in macrophage polarization towards the M1 pro-inflammatory phenotype is also found in CF patients or in Non-CF cells treated with CFTR inhibitors, suggesting a defect in the STAT-6 signalling^74^. All these evidence highlights the significant role of up regulated pro-inflammatory signalling in CF pathology, and that an intervention designed to reduce it might be potentially used as therapy in the patients. Interestingly, the inhibition of KCa3.1 channels in macrophages reduces the M1 polarization portraying KCa3.1 as a therapeutic target in CF^75^.

Although, genes coding for proteins involved in the inflammatory response have been identified as CF modifiers in humans, none of them has been related with the occurrence of meconium ileus^19^. A recent study, however, has found that the −308G>A polymorphism of the *TNF*-α gene is associated with increased risk of gastrointestinal disease in patients younger than 12 months^48^. Lymphocytes need to increase the number of K_Ca_3.1 channels in the plasma membrane for complete activation and cytokine release^76^. It will be therefore important to explore whether the severity of CF intestinal disease in humans bears any relation to the levels of expression of *KCNN4* as suggested by the results of Zielenski *et al*.^24^. Interestingly, the severity of small intestinal inflammation in patients with Crohn’s disease is diminished in association with the presence of a non-coding region polymorphism leading to lower levels of expression of *KCNN4*^77^.

In summary, our results clearly demonstrate that *Kcnn4* is a modifier gene for intestinal CF phenotype in mice. Inhibition of this channel greatly reduced the lethality of the *Cftr*^*ΔF508/ΔF508*^ mouse. The alleviation of lethality is not accompanied with an improvement in intestinal transport function. Instead there is a significant reduction of inflammatory markers, highlighting the importance of the immune system in CF pathology. Whether *KCNN4* is a modifier gene for humans affected by CF remains to be explored. We encourage the development of drugs targeting K_Ca_3.1 channels as they could be of benefit for CF patients irrespective of the *CFTR* mutation they bear.

## Electronic supplementary material

Supplementary Table 1

Supplementary Table 2

Supplementary Fig 1

Supplementary Fig 2
