# Supplementary material for: Kcnn4 is a modifier gene of intestinal cystic fibrosis preventing lethality in the Cftr-F508del mouse
Source: Sci Rep. 2018 Jun 18;8:9320. doi: 10.1038/s41598-018-27465-3 (PMC6006244; doi:10.1038/s41598-018-27465-3)
Supplement: Supplementary file 1 — Supplementary Table 1 [file 41598_2018_27465_MOESM1_ESM.docx]

*Kcnn4* is a modifier gene of intestinal cystic fibrosis preventing lethality in the *Cftr*-F508del mouse.

Amber R. Philp, Texia T. Riquelme, Pamela Millar-Büchner, Rodrigo González, Francisco V. Sepúlveda, L. Pablo Cid & Carlos A. Flores.

Sup. Table 1

| **Genotype** | **Expected (%)** | **Accumulated (%)** |
| --- | --- | --- |
| ***Kcnn4*^+/+^/*Cftr*^+/+^** | 6.25 | 6.5 |
| ***Kcnn4*^-/-^/*Cftr*^+/+^** | 6.25 | 6.9 |
| ***Kcnn4*^+/-^/*Cftr*^+/+^** | 12.5 | 12.2 |
| ***Kcnn4*^+/+^/*Cftr*^+/ΔF508^** | 12.5 | 19.1 |
| ***Kcnn4*^+/-^/*Cftr*^+/ΔF508^** | 25 | 25.2 |
| ***Kcnn4*^-/-^/*Cftr*^+/ΔF508^** | 12.5 | 11.8 |
| ***Kcnn4*^+/-^/*Cftr* ^ΔF508/ΔF508^** | 12.5 | 8.5 |
| ***Kcnn4*^+/+^/*Cftr* ^ΔF508/ΔF508^** | 6.25 | 4.9 |
| ***Kcnn4*^-/-^/*Cftr* ^ΔF508/ΔF508^** | 6.25 | 4.9 |

Mendelian expected vs accumulated percentages of animals obtained after mating of ***Kcnn4*^+/-^/**and ***Cftr*^+/ΔF508^** mice.
