# Supplementary material for: Kcnn4 is a modifier gene of intestinal cystic fibrosis preventing lethality in the Cftr-F508del mouse
Source: Sci Rep. 2018 Jun 18;8:9320. doi: 10.1038/s41598-018-27465-3 (PMC6006244; doi:10.1038/s41598-018-27465-3)
Supplement: Supplementary file 2 — Supplementary Table 2 [file 41598_2018_27465_MOESM2_ESM.docx]

Kcnn4 is a modifier gene of intestinal cystic fibrosis preventing lethality in the Cftr-F508del mouse.

Amber R. Philp, Texia T. Riquelme, Pamela Millar-Büchner, Rodrigo González, Francisco V. Sepúlveda, L. Pablo Cid & Carlos A. Flores.

Supplemental Table 2. Studies for gener-related lethality of animals from Fig 5.

From Fig 5B.

| *Cftr*^ΔF508/ΔF508^ | | | | |
| --- | --- | --- | --- | --- |
|  | Dead animals | Total animals | Ratio | z-test (P) |
| Female | 4 | 11 | 0.3636 | 0.751 |
| Male | 5 | 13 | 0.3846 |  |

| *Cftr*^ΔF508/ΔF508^/*C-kit*^W-sh/W-sh^ | | | | |
| --- | --- | --- | --- | --- |
|  | Dead animals | Total animals | Ratio | z-test (P) |
| Female | 5 | 13 | 0.3846 | 0.961 |
| Male | 2 | 7 | 0.2857 |  |

From Fig 5C.

| *Cftr*^ΔF508/ΔF508^ | | | | |
| --- | --- | --- | --- | --- |
|  | Dead animals | Total animals | Ratio | z-test (P) |
| Female | 4 | 9 | 0.4444 | 1.000 |
| Male | 8 | 15 | 0.5333 |  |

| *Cftr*^ΔF508/ΔF508^/*Stat6*^-/-^ | | | | |
| --- | --- | --- | --- | --- |
|  | Dead animals | Total animals | Ratio | z-test (P) |
| Female | 2 | 11 | 0.1818 | 0.834 |
| Male | 2 | 8 | 0.2500 |  |
