# Supplementary material for: Kcnn4 is a modifier gene of intestinal cystic fibrosis preventing lethality in the Cftr-F508del mouse
Source: Sci Rep. 2018 Jun 18;8:9320. doi: 10.1038/s41598-018-27465-3 (PMC6006244; doi:10.1038/s41598-018-27465-3)
Supplement: Supplementary file 3 — Supplementary Fig 1 [file 41598_2018_27465_MOESM3_ESM.docx]

*Kcnn4* is a modifier gene of intestinal cystic fibrosis preventing lethality in the *Cftr*-F508del mouse.

Amber R. Philp, Texia T. Riquelme, Pamela Millar-Büchner, Rodrigo González, Francisco V. Sepúlveda, L. Pablo Cid & Carlos A. Flores.


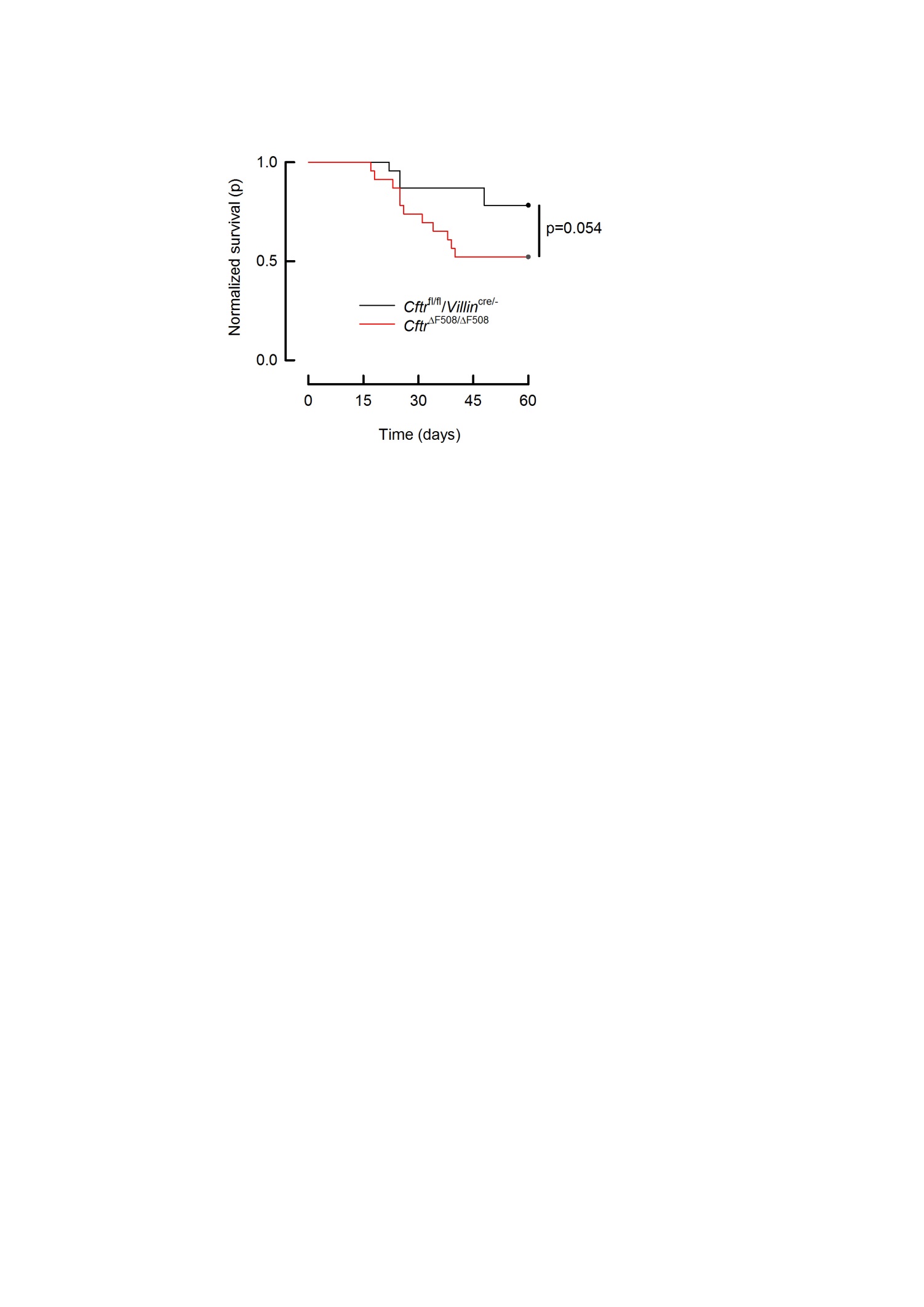
Sup. Fig 1

**Supplementary Figure 1. Conditional deletion of *Cftr* from intestinal epithelium reduces survival of mice.** Kaplan-Meier curves for the *Cftr*^fl/fl^/Villin^cre/-^ (n=23) and *Cftr*^ΔF508/ΔF508^ (n=24) animals. Percentages of survival at 60 days are 21.7% and 45.8% respectively. Log-Rank test.
