# Supplementary material for: Kcnn4 is a modifier gene of intestinal cystic fibrosis preventing lethality in the Cftr-F508del mouse
Source: Sci Rep. 2018 Jun 18;8:9320. doi: 10.1038/s41598-018-27465-3 (PMC6006244; doi:10.1038/s41598-018-27465-3)
Supplement: Supplementary file 4 — Supplementary Fig 2 [file 41598_2018_27465_MOESM4_ESM.docx]

Kcnn4 is a modifier gene of intestinal cystic fibrosis preventing lethality in the Cftr-F508del mouse.

Amber R. Philp, Texia T. Riquelme, Pamela Millar-Büchner, Rodrigo González, Francisco V. Sepúlveda, L. Pablo Cid & Carlos A. Flores.

**
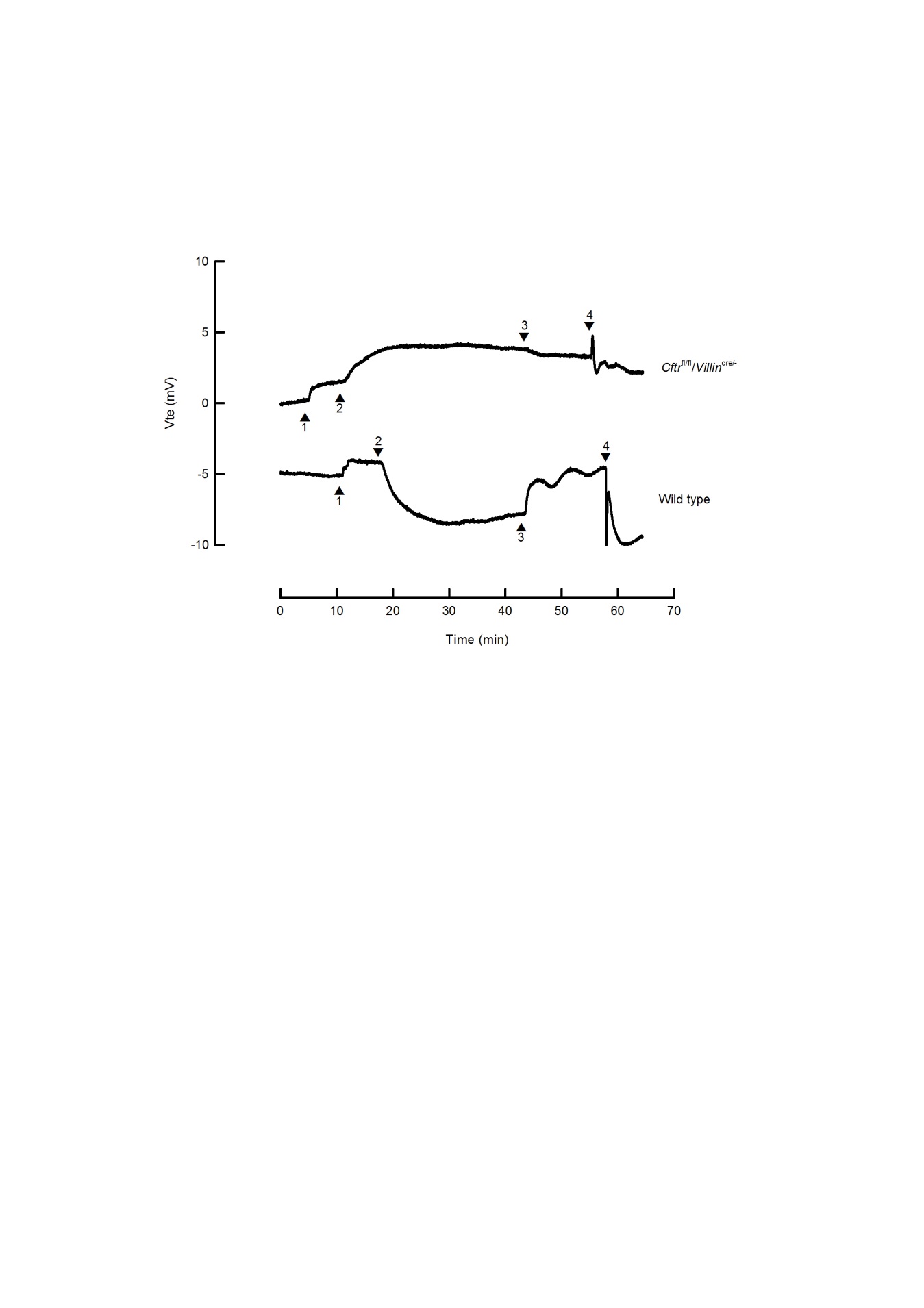
Supplemental Figure 2. The *Cftr*^fl/fl^/*Villin*^cre/-^ colon does not exhibits anion secretory responses after increasing cAMP or intracellular Ca^2+^.**

|  | Wild type (n=3) | *Cftr*^ΔF508/ΔF508^ (n=3) | *Cftr*^fl/fl^/*Villin*^cre/-^ (n=4) |
| --- | --- | --- | --- |
| Amiloride (ΔIsc) | -13.4 ± 5 | -7.7 ± 5 | -14.7 ± 6 |
| Cromanol 293B (ΔIsc) | -97 ± 17 | -10 ± 4 | 1.5 ± 14 |
| Carbachol (ΔIsc) | -229 ± 40 | 26 ± 28 | 19 ± 7 |

Representative Vte traces for wild type and *Cftr*^fl/fl^/*Villin*^cre/-^ colon. . Number 1 indicates addition of 10 µM amiloride to the apical side. Number 2 indicates the addition of cAMP-increasing cocktail (100 µM IBMX+ 1 µM forskolin) to induce cAMP-activated anion secretion that is seen as a negative negative deflection in Vte. cAMP-activated basolateral potassium channel KCNQ1/KCNE3 is inhibited with serosal 10 µM chromanol 293B (Number 3). Finally Ca2+-activated anion secretion is elicited by serosal addition of 100 µM carbachol (Number 4). All drugs are maintained constant after their corresponding addition to the bath solution. The table summarizes I_sc_ differences as described in results. Included are data for the *Cftr*^ΔF508/ΔF508^ colon for comparison.
